# Tomato (*Solanum lycopersicum*) variety discrimination and hybridization analysis based on the 5S rRNA region

**DOI:** 10.1080/13102818.2014.928499

**Published:** 2014-07-10

**Authors:** Yan-Lin Sun, Ho-Min Kang, Young-Sik Kim, Jun-Pill Baek, Shi-Lin Zheng, Jin-Jun Xiang, Soon-Kwan Hong

**Affiliations:** ^a^ School of Life Sciences, Ludong University, Yantai, Shandong, P. R. China; ^b^ Department of Horticulture, Kangwon National University, Chuncheon, Korea; ^c^ Department of Plant & Food Science, Sangmyung University, Cheonan, Korea; ^d^ Agricultural and Life Science Research Institute, Kangwon National University, Chuncheon, Korea; ^e^ Department of Bio-Health Technology, Kangwon National University, Chuncheon, Korea; ^f^ Institute of Bioscience and Biotechnology, Kangwon National University, Chuncheon, Korea

**Keywords:** tomato variety, 5S rRNA region, variety discrimination, phylogenetic relationship, genetic diversity

## Abstract

The tomato (*Solanum lycopersicum*) is a major vegetable crop worldwide. To satisfy popular demand, more than 500 tomato varieties have been bred. However, a clear variety identification has not been found. Thorough understanding of the phylogenetic relationship and hybridization information of tomato varieties is very important for further variety breeding. Thus, in this study, we collected 26 tomato varieties and attempted to distinguish them based on the 5S rRNA region, which is widely used in the determination of phylogenetic relations. Sequence analysis of the 5S rRNA region suggested that a large number of nucleotide variations exist among tomato varieties. These variable nucleotide sites were also informative regarding hybridization. Chromas sequencing of Yellow Mountain View and Seuwiteuking varieties indicated three and one variable nucleotide sites in the non-transcribed spacer (NTS) of the 5S rRNA region showing hybridization, respectively. Based on a phylogenetic tree constructed using the 5S rRNA sequences, we observed that 16 tomato varieties were divided into three groups at 95% similarity. Rubiking and Sseommeoking, Lang Selection Procedure and Seuwiteuking, and Acorn Gold and Yellow Mountain View exhibited very high identity with their partners. This work will aid variety authentication and provides a basis for further tomato variety breeding.

## Introduction

Ribosomal 5S RNA (5S rRNA) genes, which are present in multiple copies in the eukaryotic genome, are the most widely used gene family for the determination of phylogenetic relations among plant and animal species. In higher eukaryotes, 5S rRNA genes exist in tandem repeats; the number of repeats varies from less than 1000 to more than 75,000.[1,2] These genes contain the coding region and a non-transcribed spacer (NTS) region. The coding region of 5S rRNA genes is highly conserved and commonly 120 bp in length, whereas the NTS region is of different size in different species depending on the coding region and exhibits high variation. As reported in previous studies, NTS regions are very variable not only in nucleotide identity but also in sequence length in plants.[3,4] NTS lengths nearly closely vary among different loci in a genome, and high rates of nucleotide deletion in the NTS occur in some plant species.[3,5] Consequently, the 5S rRNA region, which exhibits coding region sequence conservation and high divergence within the NTS regions, has been considered a good model for studying the organization and evolution of multigene families in various plant species.[3,6,7]

Tomato (*Solanum lycopersicum*) is a major vegetable crop grown worldwide. Due to its good flavour and high nutrient value, the tomato is an economically important agricultural crop around the world.[8,9] With higher demand due to increased living standards, more tomato varieties have been bred, including Belle, Rally, Campari and Temptation. Based on their morphological and physiological characteristics, these new varieties have been determined to be distinct from all existing varieties ‘in common knowledge’ by at least one character by the relevant Variety Identification Department.[10] These tomato varieties are reported to meet established standards with respect to uniformity and stability of the characteristics used to demonstrate distinctness.[10] However, the number of registered tomato varieties remains too large to efficiently check the distinctness, uniformity and stability of their characteristics. To further discriminate tomato varieties and understand their phylogenetic relationships, we used molecular markers and DNA profiling techniques that have been widely used for the systematic identification of plants, fungi and even animals.[11,12] Molecular methods provide results that are independent of environmental influences and high levels of polymorphism. Therefore, in this study, we aimed to analyse the phylogenetic relationship of 26 tomato varieties based on sequence variation analysis of 5S rRNA genes. The observed sequence variation can elucidate phylogenetic and hybridization information.

## Materials and methods

### Plant materials

Twenty-six tomato varieties registered at the Korea Seed & Variety Service were investigated in the present study. All tomato varieties investigated in this study were provided by Kangwon National University, Chuncheon, Korea. The morphological characteristics and other traits of the tomatoes including fruit size, colour and viral resistance are shown in Table 1. Fresh mature leaves from these tomato varieties were sampled and immediately stored in liquid nitrogen until DNA extraction.

**Table 1.** Description of 26 tomato varieties including fruit shape, colour, viral resistance and NCBI GenBank accession numbers of the 5S rRNA genes.

No.	Tomato variety	Fruit shape	Fruit colour	Viral resistance	NCBI GenBank Acc. No.
(1)	Black Kiss 20	Round	Black	–	–
(2)	Black Kiss 20	Round	Black	–	–
(3)	Mini Chal	Chinese date-shaped	Red	–	–
(4)	Vitamini	Chinese date-shaped	Red	–	KF156909
(5)	Rikopin 9	Chinese date-shaped	Red	–	–
(6)	Rikopin 9	Chinese date-shaped	Red	–	KF156910
(7)	Sseommeoking	Chinese date-shaped	Red	–	KF156911
(8)	Yellow Mountain View	Chinese date-shaped	Yellow	–	KF603895
(9)	Acorn Gold	Chinese date-shaped	Yellow	–	KF603896
(10)	Gold Sugar	Chinese date-shaped	Yellow	–	KF156912
(11)	Saenggeurinbichwibol	Chinese date-shaped	Grass green	–	KF156913
(12)	Abstract Saenggeurin	Round	Grass green	–	KF156914
(13)	Seuwiteuking	Round	Red	–	KF156915
(14)	Cutie	Round	Red	–	KF156916
(15)	Rubiking	Round	Red	TY Resistance	KF156917
(16)	Ten Ten	Round	Red	–	–
(17)	Yo-Yo Captain	Round	Red	–	–
(18)	Unicorn	Round	Red	–	KF156918
(19)	Hoyong	–	Peachblow	–	KF156919
(20)	Lang selection procedure	–	Peachblow	–	KF156920
(21)	Rafito	–	Peachblow	–	KF156921
(22)	Nice Def	Round or oval	Deep red	TY Resistance	–
(23)	Max Thailang	Heart-shaped	Deep red	TY Resistance	KF156922
(24)	Madison	Quail egg-shaped	Deep red	–	–
(25)	Campari	Golf ball-shaped	Deep red	–	–
(26)	Temptation	Flat round to round	Deep red	–	–

Notes: ‘–’: undetected or undetermined. ‘TY Resistance’: Tomato yellow leaf curl virus resistance.

### Isolation of DNA, polymerase chain reaction (PCR) amplification and sequencing

DNA extractions were performed using the modified cetyltrimethylammonium bromide (CTAB) method.[13] The 5S rRNA gene was amplified using the 5SF (5′-CGGTGCATTAATGCTGGTAT-3′) and 5SR (5′-CCATCAGAACTCCGCAGTTA-3′) primer set [14] in a 20 μL polymerase chain reaction (PCR) reaction. PCR was performed using a Gene Amp 9700 PCR system (Applied Biosystems Incorporated, Warrington, Cheshire, UK) with the following reaction components: 1 μL of template DNA (approximately 1–100 ng), 10 × Ex Taq Buffer (TaKaRa Bio Inc., Japan), 200 μmol L^−1^ of each desoxynucleoside triphosphate (dNTP), 0.1 μmol L^−1^ of each primer, and 0.1 μL of TaKaRa Ex Taq (5 units μL^−1^, TaKaRa Bio Incorporated, Japan). The PCR protocol included an initial denaturation step of 94 °C for 1 min, followed by 35 cycles of denaturation at 95 °C for 1 min, annealing at 53 °C for 1 min and extension at 72 °C for 1.5 min; the reaction concluded with a final extension step at 72 °C for 5 min. The amplification products were verified by electrophoresis in a 1.0% agarose gel and purified before DNA sequence analysis, using a QIAquick PCR Purification Kit (QIAGEN, Korea) or Gel Purification Kit (QIAGEN, Korea) according to the manufacturer's instructions. Purified PCR products were then sequenced at MACROGENE Advancing through Genomics (Korea, http://dna.macrogen.com/kor/).

### Sequence editing and alignment

Sequencing results were edited and assembled using the software DNAMAN version 6.0 (Lynnon Biosoft Corporation, USA, www.lynnon.com). Analogues of the identified sequences and nucleotide sequence comparisons were detected using Basic Local Alignment Search Tool (BLAST) network services against several databases (http://www.ncbi.nlm.nih.gov/). The phylogenetic relationships were analysed based on the multiple sequence alignment of the ITS1-5.8S-ITS2 region using the software DNAMAN version 6.0. Assembled sequences were deposited in the National Center for Biotechnology Information (NCBI, http://www.ncbi.nlm.nih.gov/). The NCBI GenBank accession numbers of the tomato varieties investigated in this study are shown in Table 1.

## Results and discussion

### PCR amplification of the 5S rRNA gene

The 5SF and 5SR primers used for PCR amplification of the 5S rRNA gene are located in the middle of the highly conserved coding region and downstream of the NTS region, respectively. The sequences of the PCR products were aligned using DNAMAN version 6.0 (Figure 1). Among the samples of the 26 investigated tomato varieties, only 16 DNA sequences were amplified successfully. The remaining sequences were often contaminated with fungal and bacterial DNA, strongly affecting the PCR amplification of common genes, i.e. the 5S rRNA gene. Based on our sequencing results, the 5S rRNA genes of each tomato variety contained a partial coding region (approximately 38–54 bp) and the NTS region (approximately 229–238 bp, Figure 1). Figure 1. DNA alignment of the 5S rRNA gene sequences of 16 tomato varieties including the partial coding region and the non-transcribed spacer (NTS) region. The nucleotide arrays marked with red frames are the end sites of coding regions. The NTS region begins directly following the end site. Dots mark the deletion of a nucleotide, and short lines indicate that a nucleotide is identical to that of the reference sequence.

**Figure:**
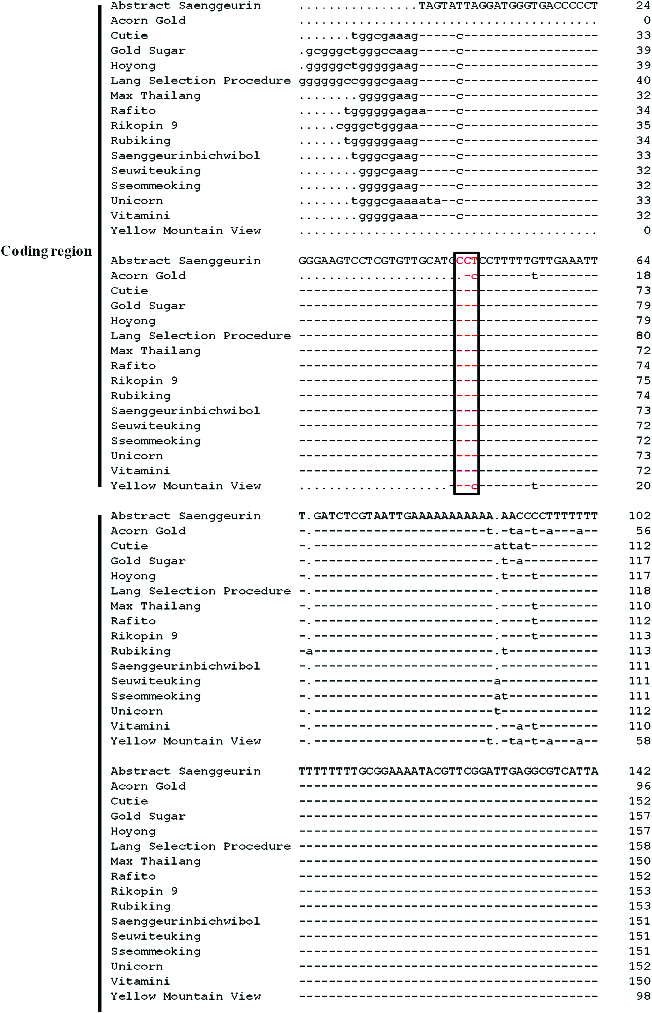

**Figure:**
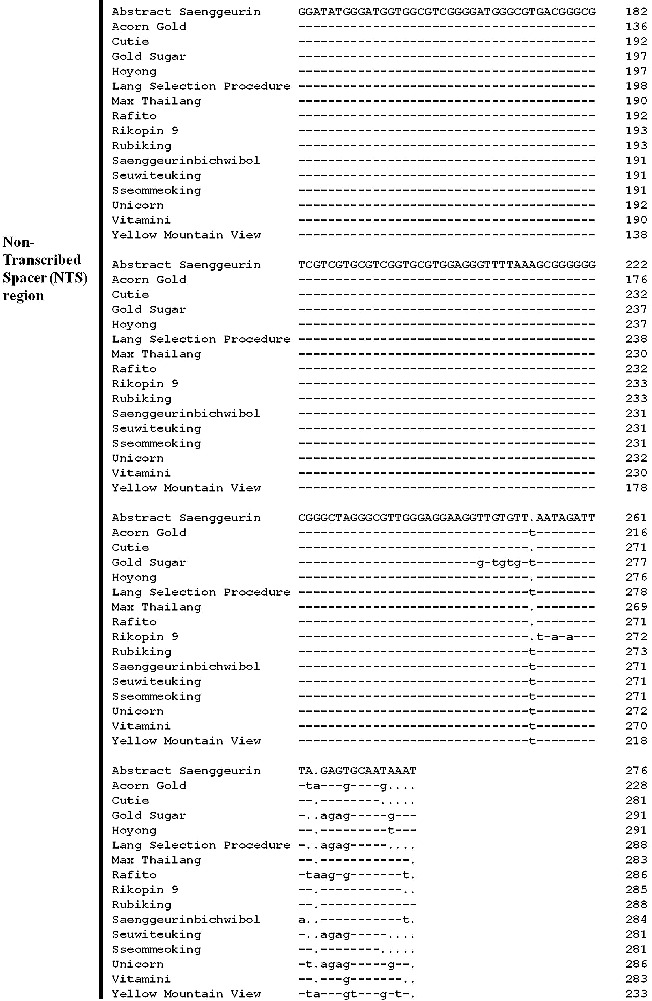


### Sequence analysis of the 5S rRNA gene

As reported previously, the 5S rRNA gene encodes a highly conserved region approximately 120 bp in length,[15] which is commonly used for more accurate sequence alignment. Non-incident shifting of nucleotides in the highly conserved coding region can be used as a reference in the sequence analysis programme. Within the 5S rRNA gene, the coding region proved to be a valuable target for the study of phylogenetic relationships due to its highly conserved sequence. Moreover, the NTS region was informative for the study of phylogenetic relationships at the interspecific and intergeneric levels due to the faster rate of divergence in comparison to the highly conserved coding region. Differences in spacer regions are generally considered to result from duplication or deletion events and are mostly accumulated in the middle spacer region.[2,7]

**Figure 2.**
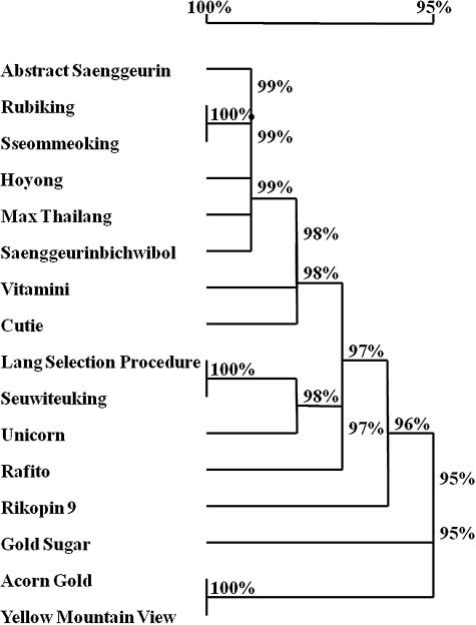
Phylogenetic tree constructed based on the 5S rRNA gene sequences of 16 tomato varieties.

In Figure 1, the red frame denotes the end site of the coding region. Excluding Abstract Saenggeurin and Yellow Mountain View, all other tomato varieties ended with CCT in the coding region of the 5S rRNA gene. All NTS regions of the tomato varieties investigated in this study started with CCT, and this nucleotide site was aligned with the start codon of the NTS of the 5S rRNA gene. A phylogenetic tree was constructed based on the sequence variation of the NTS region (Figure 2). Some tomato varieties exhibited very high identity, such as Rubiking and Sseommeoking, Lang Selection Procedure, and Seuwiteuking, Acorn Gold and Yellow Mountain View (Figure 2).

### Hybridization analysis based on the 5S rRNA gene

**Figure 3.**
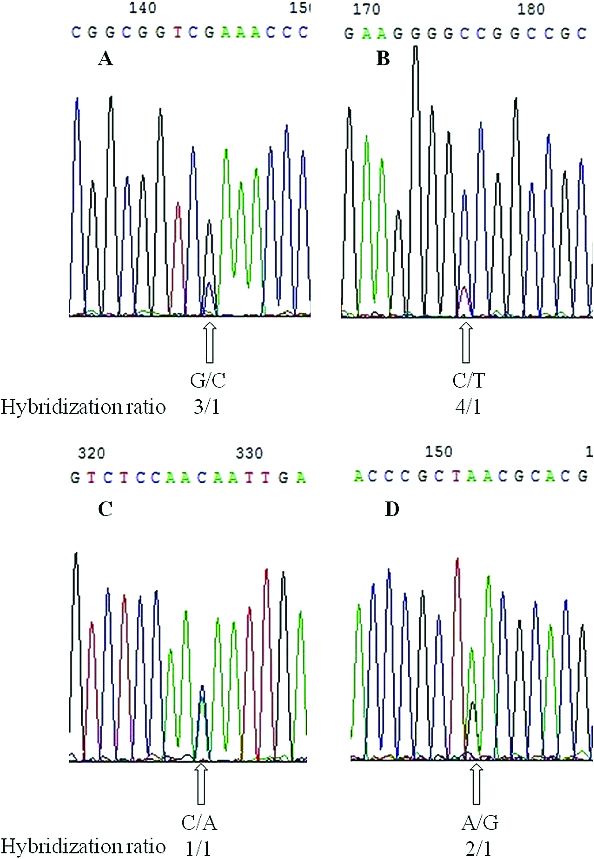
Hybridization analysis of tomato varieties based on the Chromas sequencing result. Yellow Mountain View (No. 8) and Seuwiteuking (No. 13) were used as examples. Hybridized nucleotide sites are marked with arrows under the Chromas sequencing result, and the nucleotides present and the hybridization ratio are shown below. Hybridized nucleotide sites in Yellow Mountain View sequences (A, B and C); a hybridized nucleotide site in a Seuwiteuking sequence (D).

Multiple copies of 5S rRNA gene families, as with other multigene families, can undergo concerted evolution due to homogenizing forces that lead to a high level of identity of all gene copies within the species and intraspecies. Sequence differences result from normal levels of divergence between orthologous genes in different species. These differences are associated with the number of repeats in an array, the identity of natural selection, and the effective population size,[16–18] and therefore represent hybridization information among varieties.[19,20] Our results also strongly support this model. For example, when we sampled Yellow Mountain View and Seuwiteuking tomato varieties, we found three hybridized variable nucleotide sites in the 5S rRNA gene in Yellow Mountain View but only one site in Seuwiteuking (Figure 3). The nucleotide variations were all caused by the use of different father and mother plants for variety hybridization. The occurrence of appearance of variable nucleotide sites depended on the degree of the hybridization present and the varieties used for hybridization. The degree of hybridization was apparent from the number of variable nucleotide sites and the hybridization ratio (Figure 3). Sequence variation of the NTS region of the 5S rRNA gene and the phylogenetic relationships among the tomato varieties clearly showed their homogenization, which provides a basis for tomato hybridization and breeding.

## Conclusions

In this study, we attempted to analyse the phylogenetic relationships among 26 tomato varieties based on their 5S rRNA gene sequences, although 5S rRNA gene sequences of only 16 tomato varieties were amplified and used to analyse the phylogenetic relationships. The observed sequence variation of the 5S rRNA genes also represents information regarding the hybridization of the various tomato varieties studied. This work helps to further our understanding of the phylogenetic relationships among tomato varieties and provides a basis for tomato variety breeding. 
